# Conserved recurrent gene mutations correlate with pathway deregulation and clinical outcomes of lung adenocarcinoma in never-smokers

**DOI:** 10.1186/1755-8794-7-32

**Published:** 2014-06-04

**Authors:** Zhifu Sun, Liang Wang, Bruce W Eckloff, Bo Deng, Yi Wang, Jason A Wampfler, JinSung Jang, Eric D Wieben, Jin Jen, Ming You, Ping Yang

**Affiliations:** 1 Department of Health Sciences Research, Mayo Clinic, 200 First St SW, Rochester, MN 55905, USA; 2 Department of Pathology, Medical College of Wisconsin, Milwaukee, WI 53226, USA; 3 Medical Genome Facility, Mayo Clinic, Rochester, MN 55905, USA; 4 Department of Pharmacology and Toxicology, Medical College of Wisconsin, Milwaukee, Wisconsin 53226, USA; 5 Thoracic Surgery Department, Institute of Surgery Research, Daping Hospital, Third Military Medical University, Chongqing, People’s Republic of China; 6 Division of Preventive Medicine, School of Environmental Science and Public Health, Wenzhou Medical University, Wenzhou, Zhejiang, China

**Keywords:** Lung adenocarcinoma of never smoker, Somatic mutations, Pathway deregulation, Patient survival

## Abstract

**Background:**

Novel and targetable mutations are needed for improved understanding and treatment of lung cancer in never-smokers.

**Methods:**

Twenty-seven lung adenocarcinomas from never-smokers were sequenced by both exome and mRNA-seq with respective normal tissues. Somatic mutations were detected and compared with pathway deregulation, tumor phenotypes and clinical outcomes.

**Results:**

Although somatic mutations in DNA or mRNA ranged from hundreds to thousands in each tumor, the overlap mutations between the two were only a few to a couple of hundreds. The number of somatic mutations from either DNA or mRNA was not significantly associated with clinical variables; however, the number of overlap mutations was associated with cancer subtype. These overlap mutants were preferentially expressed in mRNA with consistently higher allele frequency in mRNA than in DNA. Ten genes (*EGFR, TP53, KRAS, RPS6KB2, ATXN2, DHX9, PTPN13, SP1, SPTAN1 and MYOF*) had recurrent mutations and these mutations were highly correlated with pathway deregulation and patient survival.

**Conclusions:**

The recurrent mutations present in both DNA and RNA are likely the driver for tumor biology, pathway deregulation and clinical outcomes. The information may be used for patient stratification and therapeutic target development.

## Background

Lung cancer in never smokers becomes one of the major health burdens. Majority of primary lung cancer in never smokers is adenocarcinoma, which has distinct genetic and molecular profiles as well as demographic and clinical characteristics compared to those observed in smokers [1]. While *EGFR* (epidermal growth factor receptor) is commonly mutated in adenocarcinoma of never smoker patients, the mutations in other genes are much less common than in the smoker patients [2]. Mutations in genes such as *HER2, EML4-ALK, KRAS* and *BRAF* are all very low with mutation rate of 4.6% 4.3%, 2.0%, and 0.6%, respectively [3]. Rigorous search for new mutations and treatment targets have been carried out during the past few years with the latest next generation sequencing technology such as whole genome or targeted exome sequencing. A study of 183 lung adenocarcinomas by exome-sequencing with 27 never smokers included confirms the mutation profile differences between never smokers and smokers [4]. However, the recurrent mutations in never smokers (> = 2 samples, out of 2,528 somatic point mutations) are only detected in three genes, *EGFR, TBL3* and *HSD17B7P2* in the data. In the larger TCGA (The Cancer Genome Atlas) data with 41 never smokers, only 8 recurrent mutations (out of 1,306 point mutations) in 8 genes (*C21orf99, FAM182B, CCDC144C, FLJ36000, DDX11L2, EEF1B2, FRG1B, and EGFR*) are reported. However, the functional impacts and clinical relevance of these genes have not been further investigated. The low frequency of recurrent mutations and the little overlap of mutated genes (except EGFR) across the studies may suggest the mutational drivers for lung cancer in never smokers can be diverse and highly individualized. It is also noted that majority of mutations in cancers are random noise or passenger mutations due to a high rate of DNA replication and compromised DNA repair system, which do not play a significant role in cancer development, progression or patient outcomes. Distinguishing the driver from passenger mutations is challenging and the common approaches are to identify those genes harboring more mutations than expected given the average background mutation frequency for the cancer type. The approach can generate a long list with the sample number increasing, many of which are likely false [5]. Although this may be mitigated by incorporating mutation rate heterogeneity by a newly proposed method [5], it requires a large number of samples to be more reliable.

Most tumor somatic mutations are detected from DNA although about a third of the genes are not expressed in a particular tissue. The genes that are not expected to be expressed at all in a particular tissue or tumor (i.e., except the genes that are turned off abnormally) less likely have an important role in tumor development and progression and the mutations in these genes therefore may be less likely functional. Additionally, evidence shows that selective pressure against protein-altering mutations in expressed genes, may lead to passenger mutations more enriched in unexpressed genes as reported in a case of lung cancer [6]. For the genes with mutations in DNA, some may just be random noises not passed on to RNA to affect protein translation due to the effects of transcription-coupled repair [7]. In this study, we hypothesized that mutations present in both DNA and RNA of adenocarcinoma of the lung in never smokers were more likely functional in driving tumor biology and tested the hypothesis using 27 tumor and normal pairs with both exome-seq and mRNA-seq. We found 282 mutations in 262 genes and 10 genes with recurrent mutations. These mutated genes were highly enriched in cancer development and progression processes. The overlap mutations between DNA and mRNA were better correlated with clinical variables. More importantly the conserved recurrent mutations in the 10 genes were highly correlated with pathway deregulation, which further predicted patient clinical outcomes.

## Methods

### Experimental design

Twenty-seven lung cancer patients with stage I adenocarcinoma from never smokers were selected from the Genetics and Epidemiology of Lung Cancer Program database at Mayo Clinic. The eligible patients were those who were enrolled from 1998 to 2008 with surgery and stored fresh frozen tumor and normal lung tissues. The written consents for participating in the study were obtained and the study protocols were approved by the Mayo Clinic Institutional Review Boards. A pair of fresh frozen tissue, one from the primary tumor and another from the unaffected normal lung, was cut, stained, and reviewed by an experienced pathologist to make sure each case’s correct diagnosis and sufficient tumor component (greater than 70%) to be eligible for the study. The tumor components were marked and macro-dissected from the same tissue block for DNA and RNA extraction.

### Exome-seq and analysis

Exonic sequencing was enriched using Agilent’s SureSelect technology (version 2), which targets 51 Mb of sequence from 212,911 exons and their flanking regions in ∼ 20,000 genes. The purified capture products were then amplified using the SureSelect GA PCR primers (Agilent) for 12 cycles. Sequencing was carried out with HiSeq 2000 using 100-bp paired-end reads.

Somatic mutations between the paired tumor and normal sample for each patient were called using our customized analytical pipeline, TREAT [8]. Briefly, the raw sequence reads in FASTQ format were aligned to a human reference genome (NCBI human genome assembly build 37) using BWA [9]. The PCR duplicates were detected and removed by Picard (http://picard.sourceforge.net). Further local realignment and recalibration were conducted by GATK [10]. Single sample SNVs were called by SNVmix [11] and INDELs by GATK. Somatic mutations between the paired tumor and normal were called using VarScan 2 (version 2.2.11) [12] with following criteria: minimum coverage for normal 8, minimum coverage for tumor 6, tumor purity 0.8, somatic p value less than 0.05. The SNVs were annotated by ANNOVAR [13] and functional impacts were predicted by combination of SIFT [14] and POLYPHEN [15].

### mRNA-seq and data analysis

Total RNA extraction was performed using Exiqon’s miRCURY RNA Isolation Kit. The pair end sequencing was carried out using the Illumina HiSeq 2000 sequencer at 100 bps.

Sequence reads in fastq format were processed using our internal RNA-seq pipeline. The reads were aligned to the human genome build 37 using TopHat (1.3.3) with Bowtie (0.12.7) [16,17]. HTSeq (0.5.3p3,) was used to perform gene counting while BEDTools (2.7.1) [18] was used to count the reads mapping to individual exons. Differential gene expression was identified at raw gene count using edgeR [19]. For analyses that compared relative expression across genes or pathway deregulation analysis, log2 RPKM (reads per kilobase per million mapped reads) normalized data [20] was used.

For somatic mutations from the mRNA-seq, we first removed all reads that were not uniquely mapped to the genome and any reads that were marked as duplicates from above aligned bam file. The somatic mutations were called using VarScan2 (version 2.2.11) comparing tumor and its paired normal directly at each position with sufficient coverage with the same parameters as the exome-seq data, i.e., the minimum coverage for normal 8, minimum coverage for tumor 6, tumor purity 0.8, and somatic p value less than 0.05. The mutation calls were annotated by ANNOVAR [13].

The overlap mutations between the DNA and the RNA were found by comparing the mutations detected from each at the exact same genomic location and with the same mutated allele. Only non-synonymous mutations were kept for further downstream analyses.

### Pathway deregulation analysis by mRNA-seq data

A gene mutation may not necessarily cause its expression change. However, it may lead to abnormal interaction with other proteins in a pathway or network. Instead of gene focused analysis, we conducted pathway deregulation assessment for each tumor on 814 pathways characterized in the KEGG, REACTOME and BIOCARTA database with “pathifier” [21]. We started with the RPKM normalized gene expression data for both tumor and normal and a pathway deregulation score for each pathway in each sample was calculated using the normal samples as reference. Once the scores were obtained, we filtered out pathways with no much variation across 27 tumors and kept 272 pathways with pathway score standard deviation greater than 0.2 for unsupervised clustering, which used 1-Pearson correlation as distance matrix and centroid as linkage method. The formed sample clusters by pathway deregulation severity scores were used to correlate with clinical variables and patient survival.

### Clinical association of tumor clusters by pathway deregulation score and overlap somatic mutations

We conducted the association study between the number of somatic nonsynonymous mutations from exome-seq, mRNA-seq and the overlap between the two with tumor stage and histological type, degree of differentiation, and tumor clusters formed by pathway deregulation score using Kruskal-Wallis test. The correlation of the number of non-synonymous mutations from the above three data with the mean pathway deregulation score was performed by Pearson correlation. Kaplan-Meier survival was applied to tumor hazard clusters by pathway deregulation score; The Cox proportional model was utilized for individual gene expression or number of mutations associated with survival.

### Mutations validated by Lung/OncoCarta for selected oncogenes

Among the 27 tumors, we conducted mutation validation for 26 tumors using the combination of Lung/OncoCarta panels (http://www.sequenom.com/). Mutations found from those samples were compared with the data from exome-seq and mRNA-seq. Only single nucleotide mutations were used and compared in this study.

## Results

### Clinical and pathological characteristics of 27 never-smokers with adenocarcinoma

Among the 27 patients, 4 were male and 23 were female with average age 66.1 (+/- 12.6). There were 4 bronchi-alveolar adenocarcinomas (BAC) (reclassified as adenocarcinoma in situ according to 2011 ASLC/ATS/ERS classification) and 23 adenocarcinomas (AD). All patients were in stage I, with 10 in IA and 17 in IB. Seven patients died at the last follow-up, with average follow-up time of 5.63 years (ranging from 0.36 to 11.96 years with standard deviation of 2.64 years). Twenty tumors were well-differentiated and 7 were moderately differentiated (Table 1).

**Table 1:** Clinical and pathological information for 27 adenocarcinomas in the study

**Tissueid**	**Age**	**Sex**	**Stage**	Cell*	**Diff**^**#**^	**Grade**^**+**^	**Status**	**TTLF**
Lu1031	57	Female	IA	AD	Well	II	0	8.59
Lu1050	58	Male	IB	AD	Well	II	0	7.54
Lu1068	57	Female	IB	AD	Mod	III	1	3.28
Lu106	63	Male	IB	AD	Well	II	0	11.96
Lu113	89	Female	IA	AD	Well	II	1	5.68
Lu1182	61	Female	IA	BAC	Well	II	0	8.24
Lu1321	68	Female	IB	AD	Well	II	0	6.75
Lu1377	64	Female	IA	AD	Well	II	0	0.36
Lu1405	54	Female	IB	AD	Mod	III	0	8.03
Lu1450	74	Female	IB	AD	Well	II	0	8.08
Lu1479	64	Female	IA	AD	Well	II	0	8.08
Lu1518	55	Female	IB	AD	Mod	III	0	8.01
Lu1606	88	Female	IA	AD	Well	II	0	6.75
Lu1659	71	Female	IA	AD	Well	II	1	4.09
Lu1682	74	Female	IA	AD	Well	II	1	3.60
Lu1790	73	Female	IB	AD	Well	II	0	6.20
Lu1821	32	Male	IB	AD	Mod	III	0	6.47
Lu1848	46	Female	IB	AD	Well	II	0	5.29
Lu1942	70	Male	IB	AD	Mod	III	1	1.38
Lu1943	82	Female	IB	AD	Well	II	1	5.99
Lu2029	58	Female	IB	AD	Well	II	0	6.46
Lu2040	79	Female	IB	AD	Mod	III	0	0.78
Lu2368	58	Female	IB	AD	Mod	III	1	2.98
Lu2499	74	Female	IB	BAC	Well	I	0	5.03
Lu2502	77	Female	IA	BAC	Well	II	0	5.13
Lu346	67	Female	IA	AD	Well	II	0	5.08
Lu587	72	Female	IB	BAC	Well	I	0	5.85

*Tumor cell subtype. AD-adenocarcinoma, BAC-bronchioalveolar carcinoma (adenocarcinoma in situ according to 2011I ASLC/ATS/ERS classification). ^+^Grade – Mayo 4 level grade of differentiation. ^#^Diff – summarized tumor degree of differentiation from grade. I-II well, III- moderate, IV – Poorly/undifferentiated. TTLF – time to last follow up in years; status – 1 death, 0 alive.

### High agreement for SNVs called by exome and mRNA-seq

For each exome-seq sample, we generated an average of 168 million 100 bp paired end reads and 88% of reads were aligned to genome and 47% were aligned to the targeted regions (Agilent capture kit version 2, Additional file 1 for sequence depth, alignment statistics and average coverage for each individual sample). Very similarly for mRNA-seq, on average there were 168 million pair-end reads for each sample with alignment efficiency of 87% (Additional file 2 for sequence depth, alignment statistics, and number of genes detected with at least 1 tag and 1 RPKM for each individual sample from mRNA-seq). We first compared the SNV call result from exome-seq and mRNA-seq for each sample by SNVmix2 at the alternative allele probability cut-off of 0.8. There were 13,292 – 21,413 genomic positions where both exome-seq and mRNA-seq had SNVs, among which 99.88-99.98% had the same alternative allele or SNVs (Additional file 3). The high agreement confirmed the reliability of data and variant calling using either DNA or mRNA.

### Somatic mutations in exome-seq data

In the 27 adenocarcinoma, we detected an average of 1,274 somatic mutations per tumor (ranging from 537 to 1,841), about 25 mutations per megabase. When we limited the mutations in the coding regions, the average number of mutations in each tumor was 114 (45-322, about 2 per megabase ranging from 1 to 6), among which 77 (25-237) were nonsynonymous mutations. The number of mutations, either total number or nonsynonymous, were not associated with clinical and pathological variables (tumor subtype, stage, grade of differentiation, and age at diagnosis). There were 1,756 unique functional somatic mutations (non-synonymous or stop/gain/loss), among which 132 (7.5%) were present in 2 or more samples involving 80 genes.

### Somatic mutations in mRNA-seq data

For the mRNA-seq data, we detected an average of 957 (339-1,899) somatic single nucleotide mutations in each tumor, among which about 131 (45-341) were in the coding region and ~89 were non-synonymous (ranging from 31 to 249). Again, the number of mutations, either total number or nonsynonymous, was not significantly associated with clinical and pathological variables. Among the 27 adenocarcinomas, there were 2,198 unique functional somatic mutations (non-synonymous or stop/gain/loss), among which 167 (7.6%) were present in 2 or more samples involving 126 genes*.*

### Somatic mutations detected in both exome-seq and mRNA-seq

**Figure 1:**
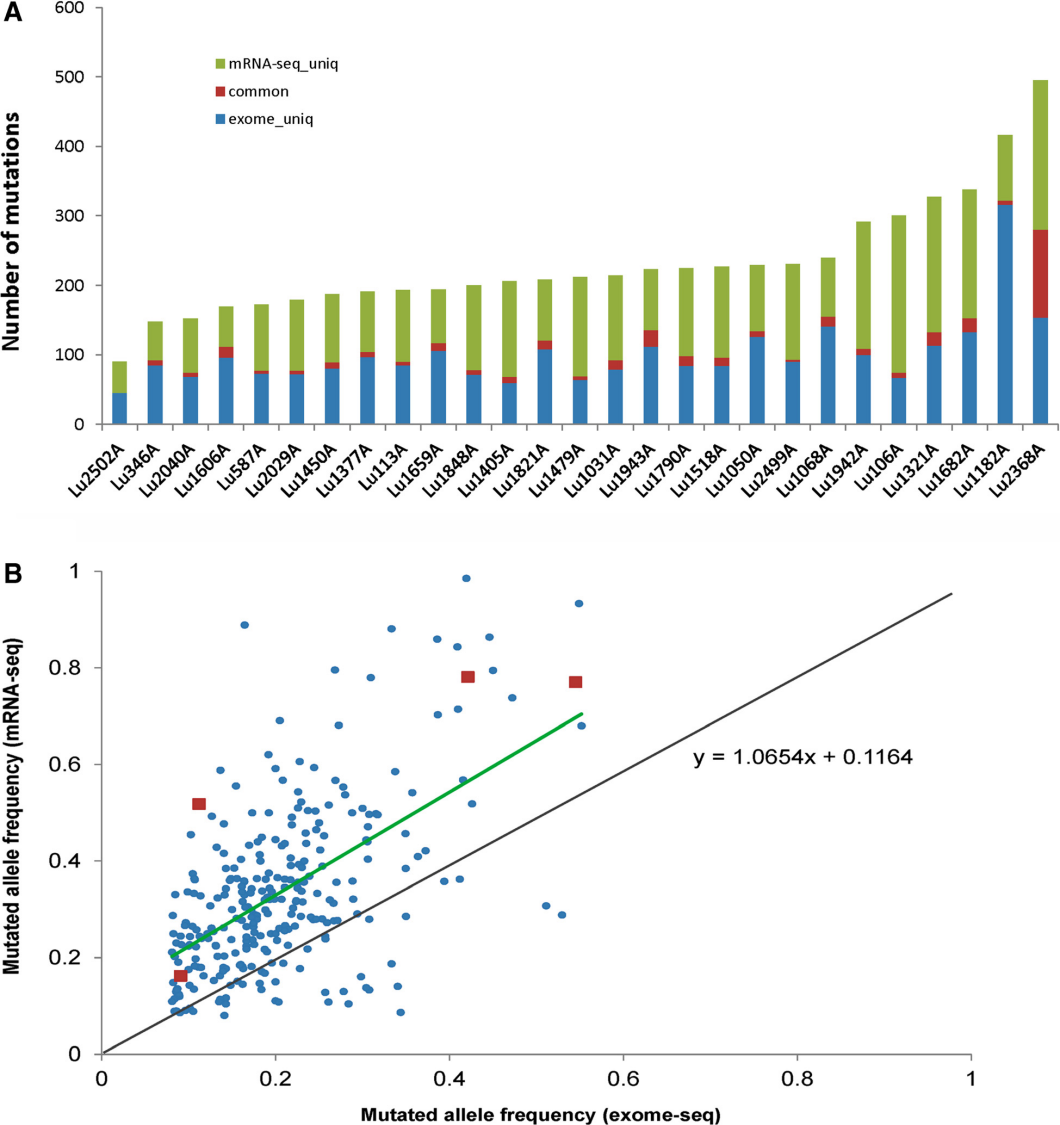
**Overlapping somatic mutations between exome-seq and mRNA-seq. A**. The number of somatic mutations from exome-seq (blue), mRNA-seq (green), and overlap between the two (red) for each tumor. **B**. Mutated allele frequency in tumor from exome and mRNA-seq. The frequency is mostly higher and is preferentially expressed in mRNA. The red dot is for *EGFR* mutation in 4 tumors, all higher in mRNA. Green line – linear trend line for the mutation allele frequency between DNA and mRNA. Black line – expected diagonal line if the frequencies were the same between DNA and mRNA.

Mutations present in both DNA and mRNA likely play a bigger role in cancer genomics, thus we focused on all coding region overlap mutations between DNA and mRNA data. Surprisingly, the comparison generated a small fraction of overlap somatic mutations between DNA and mRNA, which varied from tumor to tumor and accounted for 0-22% of RNA and 0-18% of DNA mutations (Figure 1A). These mutations (298 in total) involved 262 genes and most were nonsynonymous, with the top mutated genes as *EGFR* (4 out of 27, 14.8%), *TP53, KRAS* and *RPS6KB2* (all in 3 samples with mutation frequency 11%). Other mutated genes found in 2 samples include *ATXN2, DHX9, PTPN13, SP1, SPTAN1* and *MYOF* (Table 2, Additional file 4 for all the overlap nonsynonymous mutations). All the mutations in the 10 genes occurred at the exact same genomic location with the same mutated allele between the DNA and RNA.

**Table 2:** Recurrent mutations detected in both exome- and RNA-seq

**Gene**	**Number of samples with mutation (from 27)**	**Frequency of mutation**
*EGFR*	4	14.8%
*KRAS*	3	11.1%
*TP53*	3	11.1%
*RPS6KB2*	3	11.1%
*PTPN13*	2	7.4%
*SP1*	2	7.4%
*SPTAN1*	2	7.4%
*MYOF*	2	7.4%
*ATXN2*	2	7.4%
*DHX9*	2	7.4%

Genes with recurrent mutations. 15 out of 27 samples (55.6%) had one or more mutations in 10 genes with recurrent mutations.

Among the overlap mutations, the minimum mutated allele frequency in either DNA or mRNA was 8% (Figure 1B). For most mutations (240/282, 85%), the mutated allele frequency was higher in mRNA than in DNA, suggesting these mutations may have been preferentially expressed in mRNA transcripts. For example, *EGFR* mutation detected in 4 tumors all had a higher mutation frequency in mRNA than in DNA (Figure 1B, red highlight). Mutations in other 9 genes also had higher frequencies in mRNA except one sample for *ATXN2* and *TP53* and another for *RPS6KB2*.

### Mutations validated in Lung/OncoCarta panels

Among the 27 tumors, 26 were also tested by Lung/OncoCarta for mutations in *EGFR* and *KRAS*. All 3 *EGFR* (L858R) and 3 *KRAS* (Q61H and G12D) mutations detected in both exome-seq and mRNA-seq were validated by the assay.

### Pathway enrichment for the genes with conserved mutations

We conducted a pathway enrichment analysis for the 262 genes with mutations in both DNA and RNA using IPA (http://www.ingenuity.com/). Twenty six pathways showed a significant enrichment at p value less than 0.01. Most of these pathways were involved in cancer development or cancer processes (Additional file 5).

### Association of mutation load in DNA and RNA with clinical variables

We tested the association between the total number of nonsynonymous mutations detected in each sample, either from exome-seq or mRNA-seq, and the clinical variables of tumor stage, histological grade of differentiation, sub cell type and found no significant association for any of these variables. However, when the test was done on the number of overlap nonsynonymous mutations between exome-seq and mRNA-seq with these variables, we found a significant higher number of mutations in ADs (median of 9) than in BAC (median of 3, Kruskal-Wallis rank sum test p value = 0.004, Additional file 6).

### The 10 genes with recurrent mutations function in a closely related network

We conducted gene network analysis for the 10 genes and all but *MYOF* were mapped to the same network of “Cancer, gastrointestinal disease and respiratory disease” (Additional file 7). Although not in the same network, *MYOF* is involved in cell-to-cell signaling and interaction, cellular and embryonic development. A recent data shows high expression of this gene is associated with tumor mesenchymal transformation and aggressive phenotype [22].

### Pathway deregulation scores are correlated with mutation patterns of the 10 genes with recurrent mutations present in both DNA and RNA and clinical outcomes

**Figure 2:**
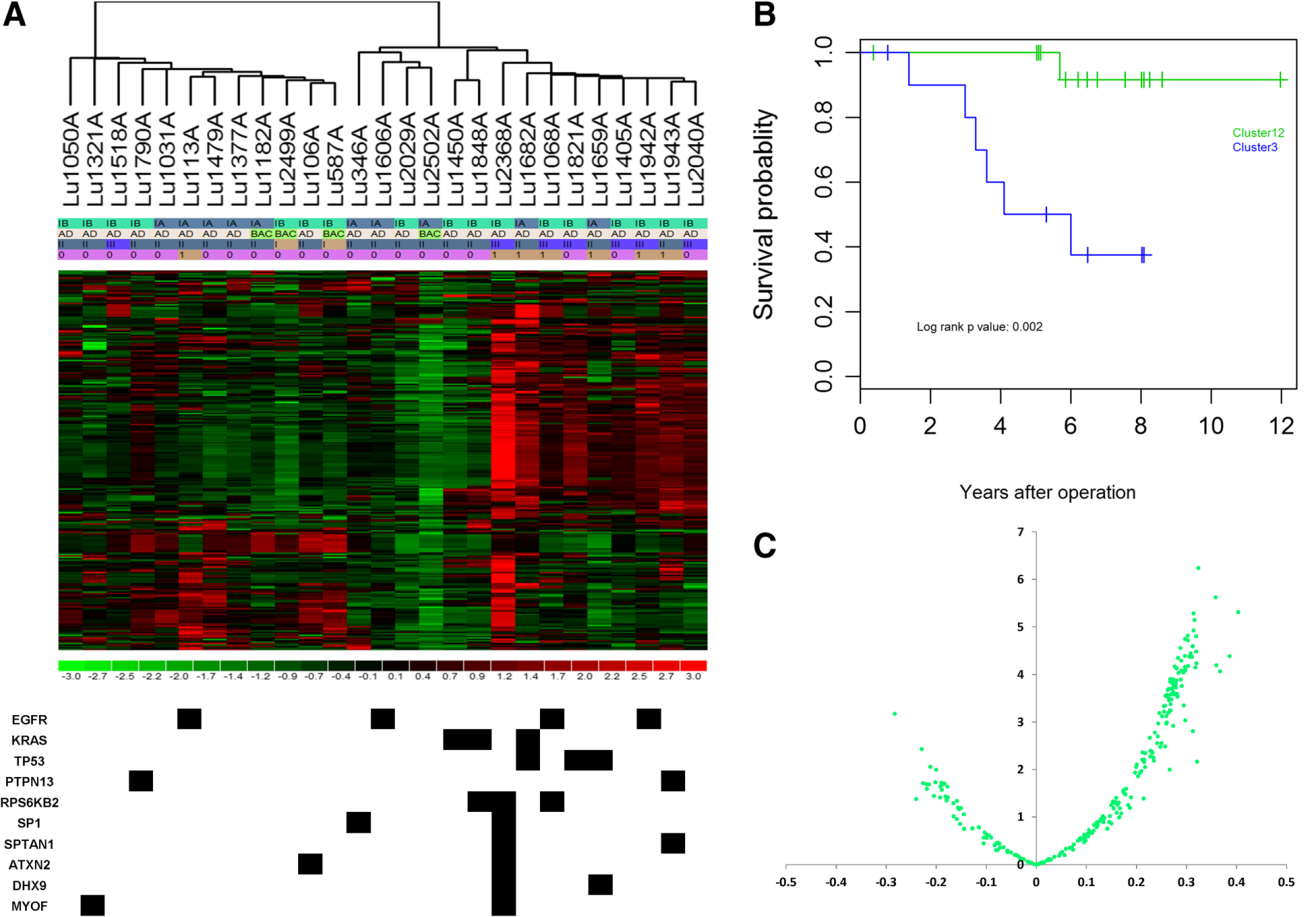
**Association of pathway deregulation with mutation profiles and patient survival. A**. Unsupervised clustering for tumors by pathway deregulation score and their association with 10 genes with recurrent mutations. **B**. Kaplan Meier survival curve by sample cluster defined by pathway deregulation score comparing Cluster 3 vs. Cluster 1, 2. **C**. Differentially deregulated pathways between patients with bad (cluster 3) and those with good survivals (cluster 1 and 2). X-axis: mean pathway score difference. Y-axis: negative log10 p value. The samples with bad survival had more pathways with a higher deregulation score.

Unsupervised clustering using pathway deregulation scores of 272 pathways showed that the tumors formed 2 main clusters (Figure 2A), the left branch with 12 samples (referred as Cluster 1) and the right with 15 samples. The right cluster could be further separated into two subclusters, one in the middle (Cluster2) with 4 samples and another on the right with 11 samples (Cluster 3). The Cluster 3 had a higher proportion of moderately differentiated tumors (Fisher’s test p value 0.01) and a higher proportion of patients who died of the disease (Fisher’s p value 0.01). The cluster also had a higher number of mutations (Fisher’s test p value 0.01) in the 10 genes (*EGFR, KRAS, TP53, PTPN13, RPS6KB2, SP1, SPTAN1, ATXN2, DHX9, MYOF*) with recurrent mutations in both DNA and RNA. No association with patient survival was found for patient age at lung cancer diagnosis, sex, tumor stage (stage IA vs IB), histological cell subtype (AD vs. BAC), histologic grade of differentiation. However, the tumor clusters formed by pathway deregulation scores were significantly associated with patient survival, either by combined Cluster 1, 2 vs. Cluster 3 (log rank p value 0.002, Figure 2B), or among three clusters (p = 0.01). The tumors in Cluster 3 had significantly a higher pathway deregulation score for majority of pathways than tumors in Cluster 1 and 2 (shown in red in Figure 2A, positive score difference in Figure 2C). The tumors with mutations in any of the 10 genes rendered worse survival than the tumors without mutations (log rank p value 0.02, Additional file 8). With the increase of mutations in these genes, the risk of death increased 2.6 fold (hazard ratio 2.55 with p value 0.004).

### Pathway mean deregulation score positively correlated with the number of mutations in each sample

**Figure 3:**
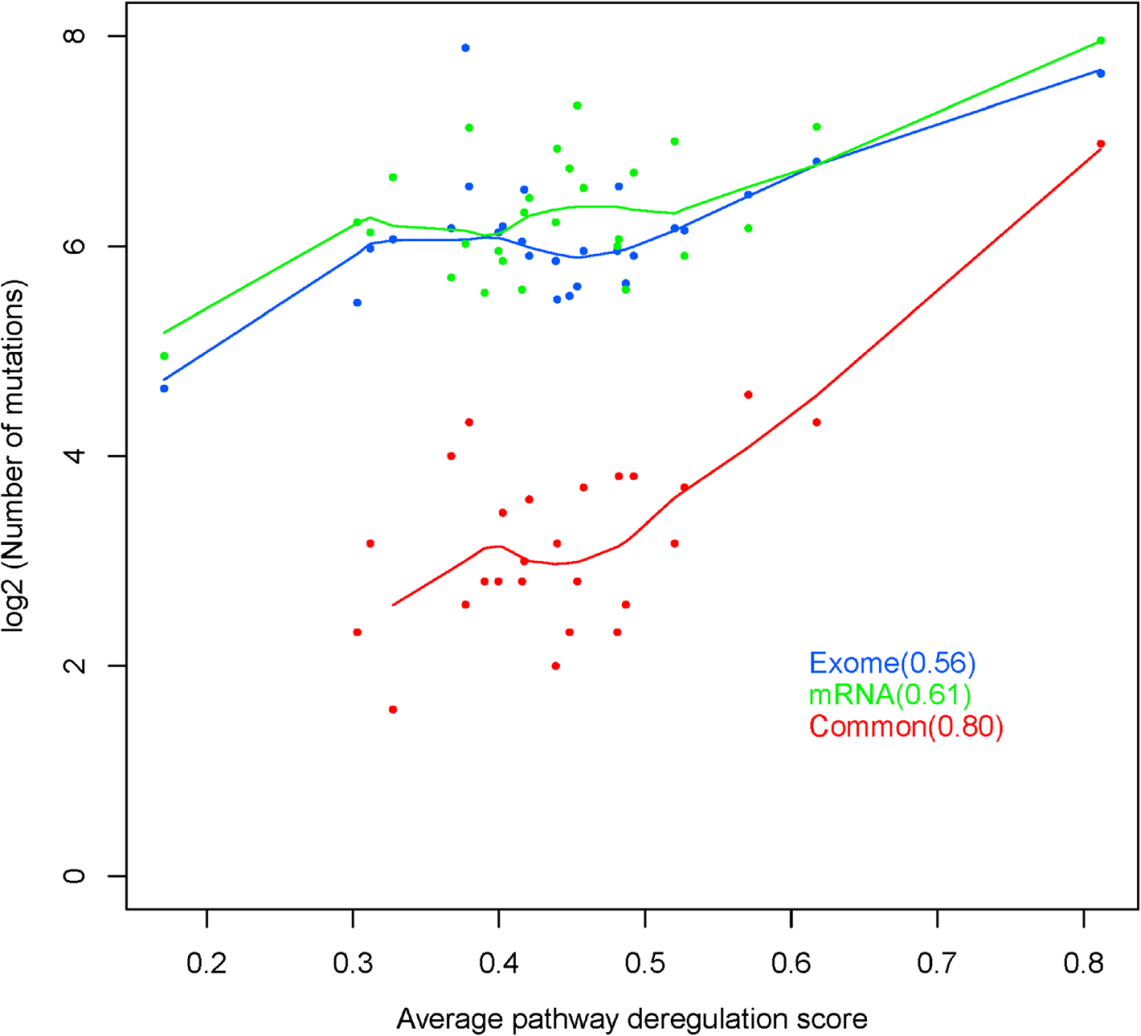
**Correlation of average pathway deregulation score and number of exome, mRNA, and overlap mutations.** The number of overlap mutations in each tumor is highly correlated with average pathway deregulation score with a correlation coefficient 0.80 although the number of mutations in either DNA or RNA is also moderately correlated with pathway deregulation.

We conducted a correlation analysis between the number of nonsynonymous mutations detected from exome-seq, mRNA-seq, and the overlap between the two and found that all were positively correlated with the mean pathway deregulation score with correlation coefficient of 0.56, 0.61 and 0.80, respectively (Figure 3). The highest correlation between the average pathway deregulation score and the overlap mutations between DNA and RNA suggested that the conserved mutations have more functional impacts on pathway regulation and network interaction.

### Expression pattern of the 10 recurrent genes in tumor and normal lung and tumors with or without mutation

Compared to the normal lungs, *RPS6KB2, TP53, EGFR, DHX9, and MYOF* were elevated and *SPTAN1, ATXN2, and SP1* were reduced in tumors. No significant change was observed for *KRAS* and *PTPN13* (Additional file 9). When comparing the gene expression of the tumors with or without mutations for each of the 10 genes, *KRAS* and *DHX9* showed higher expression (1.5 fold) in the tumors with the mutation compared to those without the mutation (Additional file 10). There was no expression difference for other genes.

### Gene expression patterns of the 10 recurrent genes in tumors with bad and good outcomes

Comparing the tumors in Cluster 3 (with bad outcome) with the tumors in Cluster 1 and 2 (good outcome), *PTPN13* was significantly down expressed with 2.15 fold change while *KRAS, SP1*, and *DHX9* were up-expressed. No significant change was seen for other genes (Additional file 11). In the Cox regression of survival analysis, *PTPN13* and *DHX9* were significantly associated with survival with a hazard ratio of 0.28 (p value 0.02) and 13.3 (p value 0.04), respectively. The higher expression of *PTPN13* predicted a better survival and the reverse was for *DHX9. KRAS* and *SP1* were borderline significant (HR 4.7, p 0.07 and 4.9, p 0.09, respectively), which both were associated with poor survival when highly expressed. No association was observed for other 4 genes.

## Discussion

Several exome or whole genome sequencing studies have been conducted on lung adenocarcinoma with a small number of never smokers included [4,7] with the aim to find high impact and recurrent mutations. However, results so far have not yielded significant progress. Most of the reported mutations are either from a few genes reported previously or the recurrent mutations across samples are very low. In 27 adenocarcinomas of never smokers [4], only three genes, *EGFR, TBL3* and *HSD17B7P2,* occurred in two or more samples. Even in the larger dataset with 41 never smokers from TCGA, only 8 genes are found with recurrent mutations (*C21orf99, FAM182B, CCDC144C, FLJ36000, DDX11L2, EEF1B2, FRG1B, and EGFR; the level II somatic mutation data for lung adenocarcinoma was downloaded from TCGA at*http://cancergenome.nih.gov*in February 2013*). Surprisingly, only *EGFR* is the gene in common across the studies and the biological relevance of other genes is little known. Those studies are all performed on DNA and no comparison is made with mutations at RNA and how the mutations can interplay with RNA expression and transcriptome network and pathway regulation remains a question. As mutations detected from either DNA or mRNA can contain artifacts from sequence or alignment errors and passenger mutations are more enriched in unexpressed genes [6], we hypothesized that mutations present in both DNA and RNA or conserved mutations were more likely legitimate and driver mutations for tumor biology.

As expected, hundreds to thousands of somatic mutations were detected at either exome or RNA sequencing for each tumor. However, the number of mutations from either was not associated with any clinical variables. The mutations present in both DNA and mRNA were only from tens to hundreds in each tumor. Collectively, these mutations involved 261 genes and they were almost all in the coding region and nonsynonymous. These genes were highly enriched in tumorigenesis and cancer related pathways and networks while the genes with functional mutations from either exome-seq (1,267 genes) or mRNA-seq (1,610 genes) were more related to non-cancerous pathways (Additional files 12 and 13). More importantly, we found 10 recurrently mutated genes were highly concentrated in the group of tumors with more deregulated pathways and worse survival. In addition to the commonly mutated genes such as *EGFR, KRAS, and TP53*, we found several genes not reported before and all involved in tumor biology. *PTPN13* a tumor suppressor is often inactivated in non-small cell lung cancer due to the loss of either mRNA and protein expression or somatic mutation [23,24]. In this set of tumors, all from never smokers, we did not see the significant differential expression compared to their paired normal lung. However, the tumors with lower expression of this gene had shorter survival than the tumors with higher expression. *RPS6KB2* (ribosomal protein S6 kinase beta-2) is a member of serione/theonine kinases which regulate protein synthesis and cell proliferation. The function gain of this gene is reported in a subset of breast cancer, which is associated with worse prognosis [25]. The genomic variants of this gene are also found to be associated with the development of colon cancer [26]. The up-regulation of this gene in lung adenocarcinoma suggests its role in lung adenocarcinoma. *SP1* is a transcription factor that regulates gene expression and may promote tumor progression and lead to bad outcome for patients as observed in our data. It may be a potential treatment target for prostate cancer [27]. *DHX9* (DEAH (Asp-Glu-Ala-His) box helicase 9) is an enzyme that catalyzes the ATP-dependent unwinding of double-stranded RNA and DNA-RNA complexes and functions as a transcriptional regulator. The higher expression of this gene is associated worse outcome in this set of patients. *ATXN2* (ataxin 2) mutation is associated with spinocerebellar ataxia type 2 and a recent study shows that the methylation of this gene is one of the nine genes that define clear-cell subtype of ovarian cancer. *MYOF* (myoferlin) depletion in breast cancer cells has shown to promote mesenchymal to epithelial transformation and stall invasion [22] with potential as a biomarker or drug target for metastatic cancer diagnosis and therapy [28]. This gene is up-regulated in lung adenocarcinoma and supports its role in tumor transformation. And finically, *SPTAN1* (spectrin, alpha, non-erythrocytic 1) belongs to a family of filamentous cytoskeletal proteins that function as essential scaffold proteins that stabilize the plasma membrane and organize intracellular organelles. This gene is implicated in DNA repair and cell cycle regulation. The reduced expression in tumors may indicate its compromised DNA repair function.

One of the strengths of our work is, instead of gene level analysis, we used pathway deregulation scores for each sample. This not only significantly reduced the dimensionality of the data but also put genes into functional context as mutations in some genes may not necessarily affect their own expression but disrupt protein-protein interaction and pathway functions. As shown, the pathway deregulation score was the best predictor for the tumor behavior and these pathway dysfunctions may be contributed by tumor somatic mutations occurred in both DNA and RNA. Either the average pathway deregulation score or the number of conserved high impact mutations can better predict patient outcomes. This may be potentially used for clinical patient stratification.

We observed the high variability of the number of overlap mutations between DNA and mRNA from tumor to tumor and examined whether it was the result of varied sequence depths. As this is dictated by the sample with the lowest sequence depth, we plotted the relationship of this with the number of overlap mutations and did not see any correlation (Additional file 14), suggesting it was not due the sequence depth difference but more likely caused by the degree of tumor abnormality. For the “mutations” called by mRNA-seq but not exome-seq in the coding region, we found about 7% were not covered or below the coverage threshold for somatic mutation call in exome-seq; 10% were called as “germline” mutations; 66% without variants in both tumor and normal (or with reference allele); and 4% as “somatic” but not reaching the significant cut-off of p value < 0.05. Unsurprisingly, for the “somatic mutations” called by exome-seq but not RNA-seq, over 46% were not or below the coverage threshold (genes not expressed); 42% as reference calls; 1% as “germline”, and 2% as “somatic” but not significant at the threshold cut-off (Additional file 15).

Our focus in this study was single nucleotide mutations. It is likely that insertions/deletions (indels) also contribute to the clinical phenotypes of the tumors. However, indel detection is more challenging for mRNA-seq data. Even for exome-seq data, longer indels are often missed by commonly used algorithms. Additionally, indels rarely occur in the same positions and the “recurrent” indels are hard to define. The functional impacts from indels are also difficult to predict. Along with copy number changes and structural variants, further research will definitely extend our understanding of the complex tumor biology of adenocarcinoma in never smokers.

## Conclusions

In summary, we identified highly clinical relevant recurrent mutations in 10 genes from never-smoker adenocarcinoma that are associated with pathway deregulation, tumor phenotype and patient clinical outcomes. These mutations are conserved and preferentially expressed in RNA and are likely the driver for tumor biology and may potentially be used for outcome prediction and treatment targets.

## Competing interests

The authors declare no conflict of interest.

## Authors’ contributions

ZS conceived and designed the study, analyzed and interpreted the data, and drafted the manuscript. LW designed the study and generated the data. BWE generated and analyzed the sequencing data. BD, YW and JAW generated and analyzed the data. JSJ conducted LungCarta mutation validations. EDW designed the study and generated the sequencing data. JJ designed the study and conducted mutation validations. MY conceived and designed the study and generated the sequencing data. PY conceived and designed the study, collected patient and samples, and interpreted the data. All authors read and approved the final manuscript.

## Pre-publication history

The pre-publication history for this paper can be accessed here:

http://www.biomedcentral.com/1755-8794/7/32/prepub

## Supplementary Material

Additional file 1 Sequence depth, alignment statistics and average coverage for exome-seq samples. Click here for file

Additional file 2 Sequence depth, alignment statistics, and number of genes detected for mRNA-seq samples. Click here for file

Additional file 3 **SNV agreement between exome-seq and mRNA-seq.** The number on the left Y axis is the number of sites commonly called by both and blue bar marks the number for each sample (both tumor and its pair normal are plotted for a total of 54 samples; only tumor sample name is shown on X axis. The normal is right after paired tumor). The number on the right Y axis is the percentage of agreement of SNV alternative allele calls between exome and mRNA-seq with the brown line for each sample. Click here for file

Additional file 4 **Overlap mutations between DNA and mRNA.** The full list of overlap mutations between DNA and mRNA. Click here for file

Additional file 5 **Enriched pathways for the genes with overlap mutations.** List of enriched pathways using the genes with mutations found in both DNA and RNA. Click here for file

Additional file 6 **Overlap mutations by cell type.** “BAC” has fewer mutations than adenocarcinoma. Y-axis. The number of overlap mutations in log10 scale. Click here for file

Additional file 7 **The 10 recurrently mutated genes in a closely related interaction network.** The 10 genes but MYOF with recurrent mutations are mapped to the same network of “Cancer, gastrointestinal disease and respiratory disease”. Click here for file

Additional file 8 **Kaplan-Meier survival curve for tumors with or without mutations in 10 genes with recurrent mutations.** Tumors with mutations in any of the genes have poor survival than those without mutations. Click here for file

Additional file 9 **Differential expression between tumor and normal for the genes with recurrent mutations from edgeR.** logFC – log2 fold change; FC – fold change; logCPM – normalized average expression across tumor and normal sample in log2 scale; PValue – differential p value between tumors and normal samples; FDR – false discovery rate. Click here for file

Additional file 10 **The expression profiles of the 10 genes in 27 tumors.** Bar graph for the gene expression of 10 genes with recurrent mutations. X-axis – sample; y-axis – log2 RPKM expression. The black bars are for sample with mutation. Click here for file

Additional file 11 **10 gene expression in tumors with good and bad outcome.** The boxplot of expression of 10 genes with recurrent mutation in the tumors of clusters 1, 2 (good outcome) and cluster 3 (bad outcome), respectively. Y-axis is the log2 RPKM expression. Click here for file

Additional file 12 Enriched pathways for the genes with functional somatic mutations from exome-seq. Click here for file

Additional file 13 Enriched pathways for the genes with functional somatic mutations from mRNA-seq. Click here for file

Additional file 14 **Sequence depth vs. number of overlap mutations between DNA and mRNA.** No correlation is observed and the tumor with the highest number of common mutations (Lu2368A) has much lower sequence depth than most of samples. X-axis is the minimum depth of tumor and normal from both exome-seq and mRNA-seq. Y-axis is the number of overlap mutations. Click here for file

Additional file 15 **Backfill information of mutation positions of one data in another.** For the mutations called in DNA (or RNA) but not in RNA (or DNA), the coverage and allele information for the genomic positions in RNA (or DNA) are examined for potential genotype/mutation calls and tabulated by category. For DNA mutations not called in RNA, majority of them (46%) are lack of sufficient coverage (genes not expressed) while majority of mutations called in RNA but not in DNA (63%) do not have the mutation allele in the tumor DNA (or reference call). Click here for file
