# Erratum to: Conserved recurrent gene mutations correlate with pathway deregulation and clinical outcomes of lung adenocarcinoma in never-smokers

**DOI:** 10.1186/s12920-016-0237-y

**Published:** 2017-01-05

**Authors:** Zhifu Sun, Liang Wang, Bruce W. Eckloff, Bo Deng, Yi Wang, Jason A. Wampfler, JinSung Jang, Eric D. Wieben, Jin Jen, Ming You, Ping Yang

**Affiliations:** 1 Department of Health Sciences Research, Mayo Clinic, 200 First St SW, Rochester, MN 55905 USA; 2 Department of Pathology, Medical College of Wisconsin, Milwaukee, WI 53226 USA; 3 Medical Genome Facility, Mayo Clinic, Rochester, MN 55905 USA; 4 Department of Pharmacology and Toxicology, Medical College of Wisconsin, Milwaukee, Wisconsin 53226 USA; 5 Thoracic Surgery Department, Institute of Surgery Research, Daping Hospital, Third Military Medical University, Chongqing, People’s Republic of China; 6 Division of Preventive Medicine, School of Environmental Science and Public Health,Wenzhou Medical University, Wenzhou, Zhejiang China

## Erratum

Data availability for article [1]:

The raw sequence data have been deposited into European Genome-phenome Archive (EGA) with accession number EGAS00001000776 =>. https://www.ebi.ac.uk/ega/home.

The raw sequence data have been deposited into Gene Expression Omnibus (GEO) with accession number GSE87340. https://www.ncbi.nlm.nih.gov/geo/)
