# Supplementary figures and images for: Conserved recurrent gene mutations correlate with pathway deregulation and clinical outcomes of lung adenocarcinoma in never-smokers
Source: BMC Med Genomics. 2014 Jun 4;7:32. doi: 10.1186/1755-8794-7-32 (PMC4060138; doi:10.1186/1755-8794-7-32)

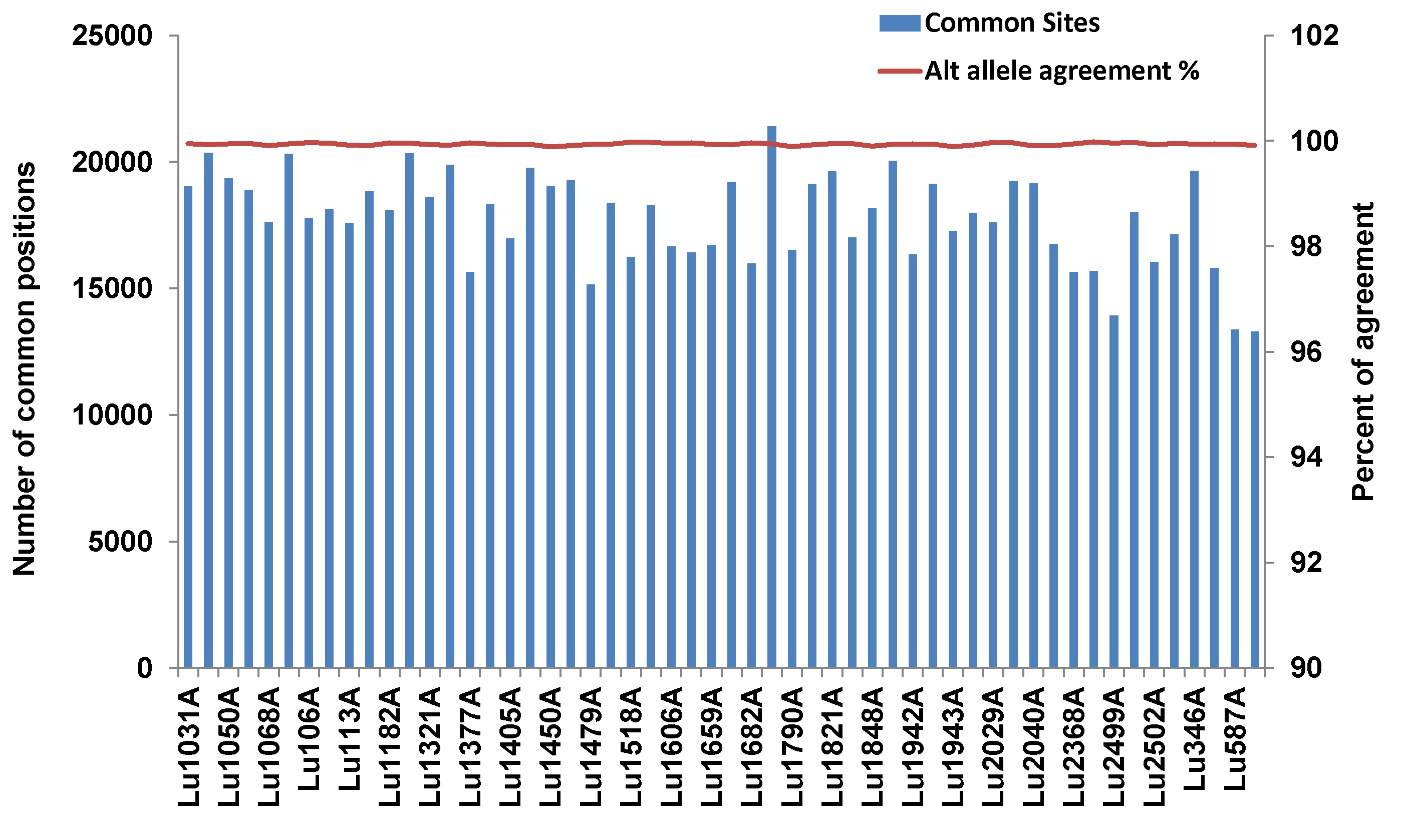

Supplement: Additional file 3 — SNV agreement between exome-seq and mRNA-seq. The number on the left Y axis is the number of sites commonly called by both and blue bar marks the number for each sample (both tumor and its pair normal are plotted for a total of 54 samples; only tumor sample name is shown on X axis. The normal is right after paired tumor). The number on the right Y axis is the percentage of agreement of SNV alternative allele calls between exome and mRNA-seq with the brown line for each sample. [file 1755-8794-7-32-S3.tif]

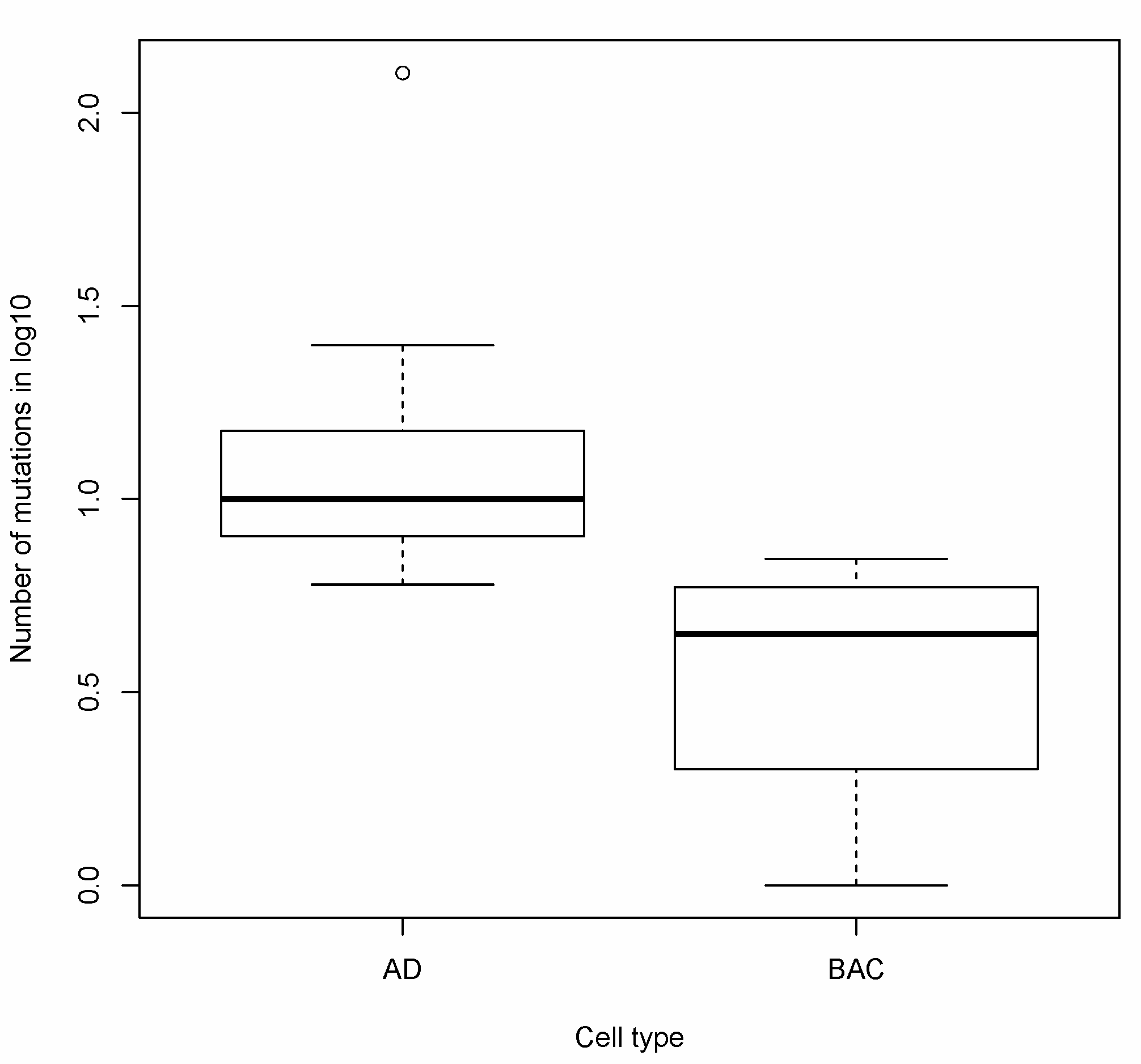

Supplement: Additional file 6 — Overlap mutations by cell type. “BAC” has fewer mutations than adenocarcinoma. Y-axis. The number of overlap mutations in log10 scale. [file 1755-8794-7-32-S6.tiff]

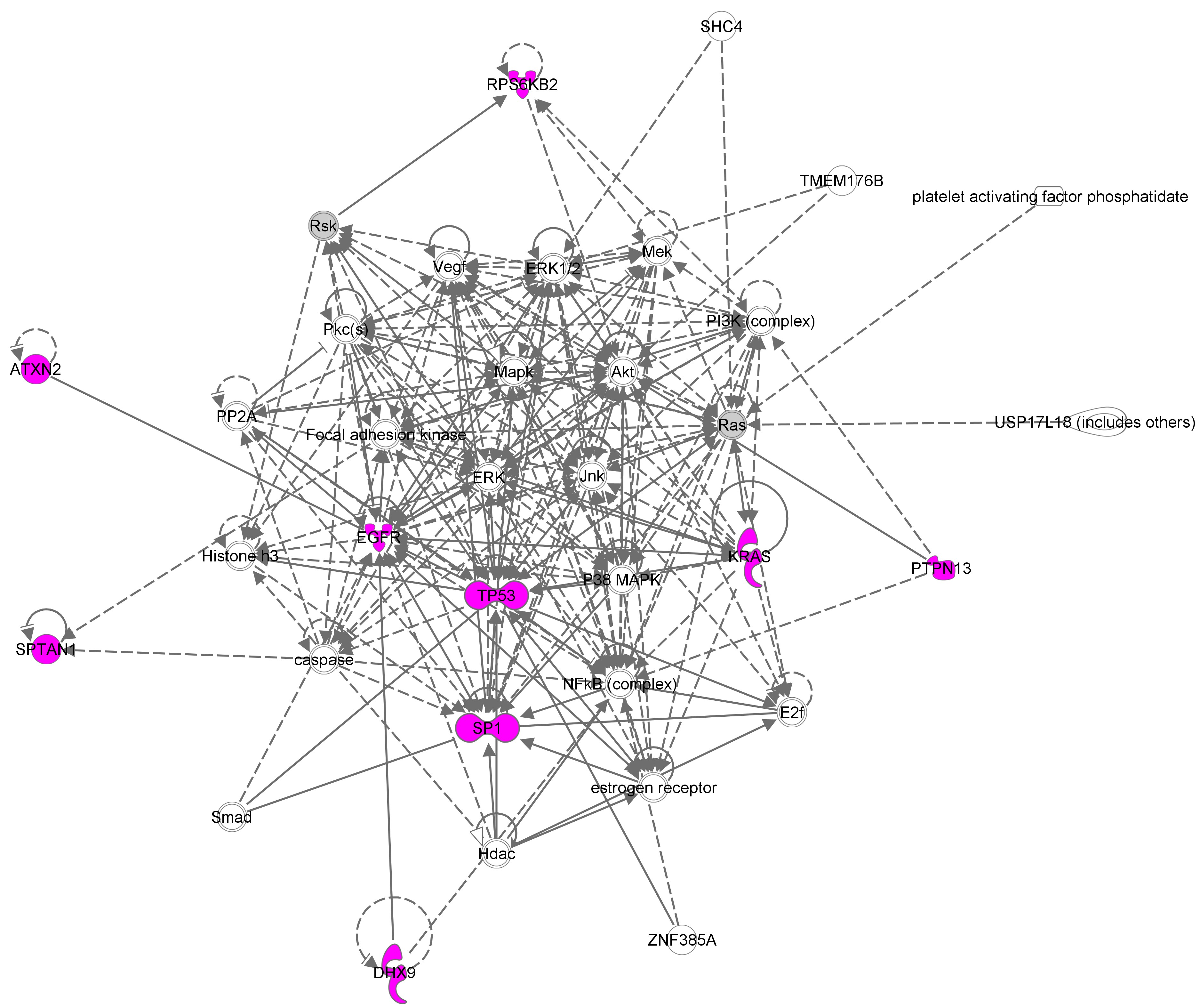

Supplement: Additional file 7 — The 10 recurrently mutated genes in a closely related interaction network. The 10 genes but MYOF with recurrent mutations are mapped to the same network of “Cancer, gastrointestinal disease and respiratory disease”. [file 1755-8794-7-32-S7.tiff]

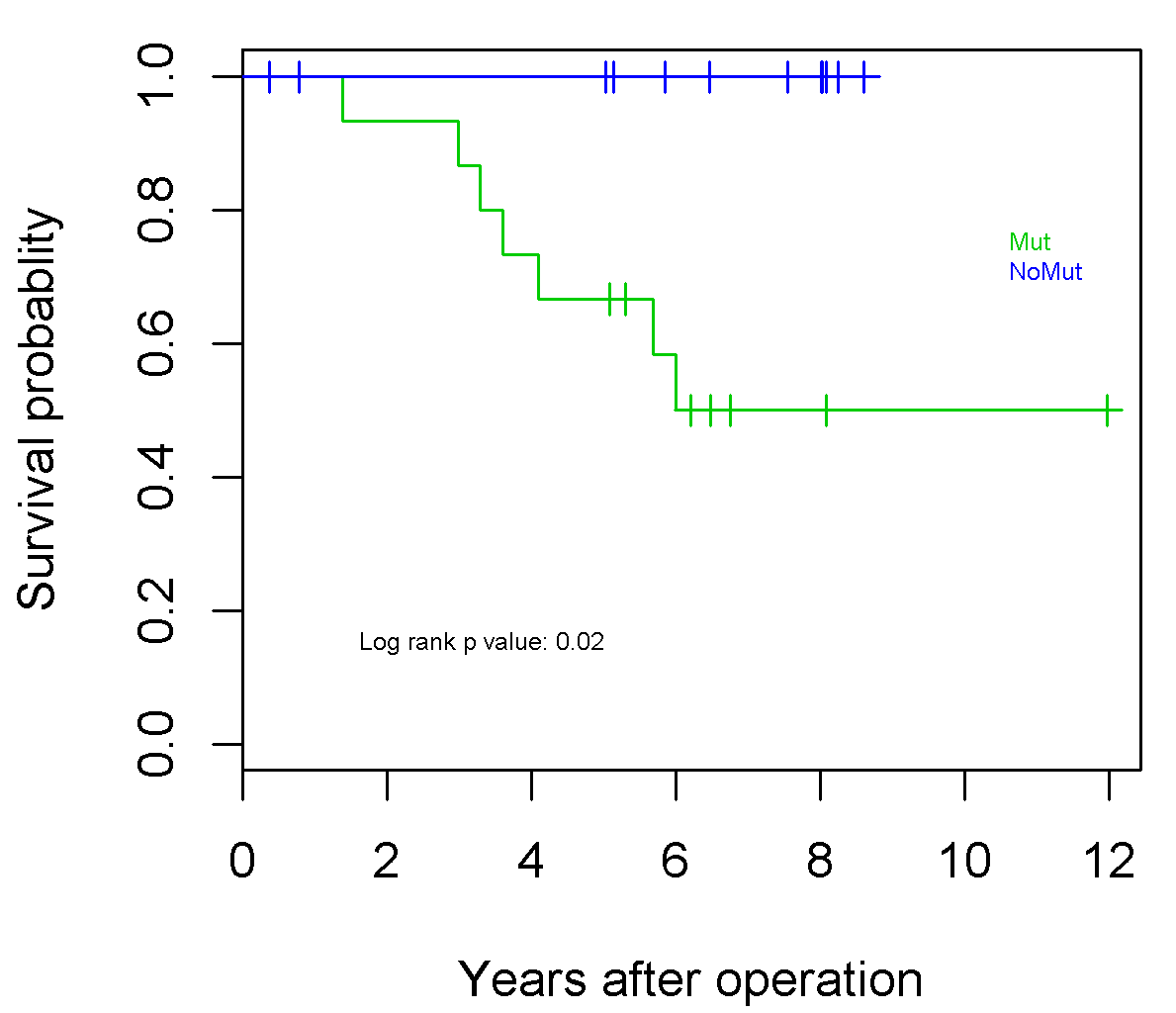

Supplement: Additional file 8 — Kaplan-Meier survival curve for tumors with or without mutations in 10 genes with recurrent mutations. Tumors with mutations in any of the genes have poor survival than those without mutations. [file 1755-8794-7-32-S8.tiff]

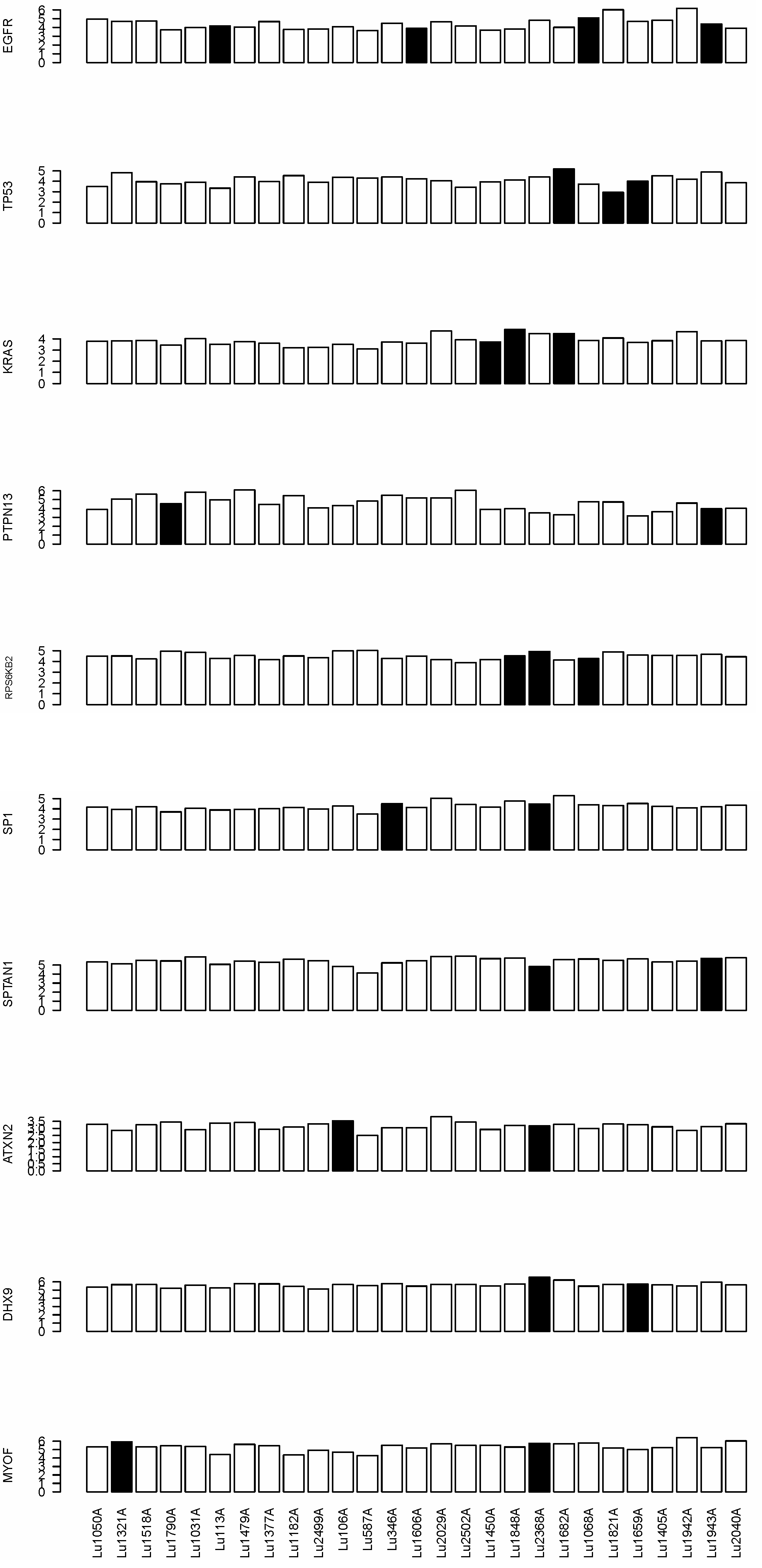

Supplement: Additional file 10 — The expression profiles of the 10 genes in 27 tumors. Bar graph for the gene expression of 10 genes with recurrent mutations. X-axis – sample; y-axis – log2 RPKM expression. The black bars are for sample with mutation. [file 1755-8794-7-32-S10.tiff]

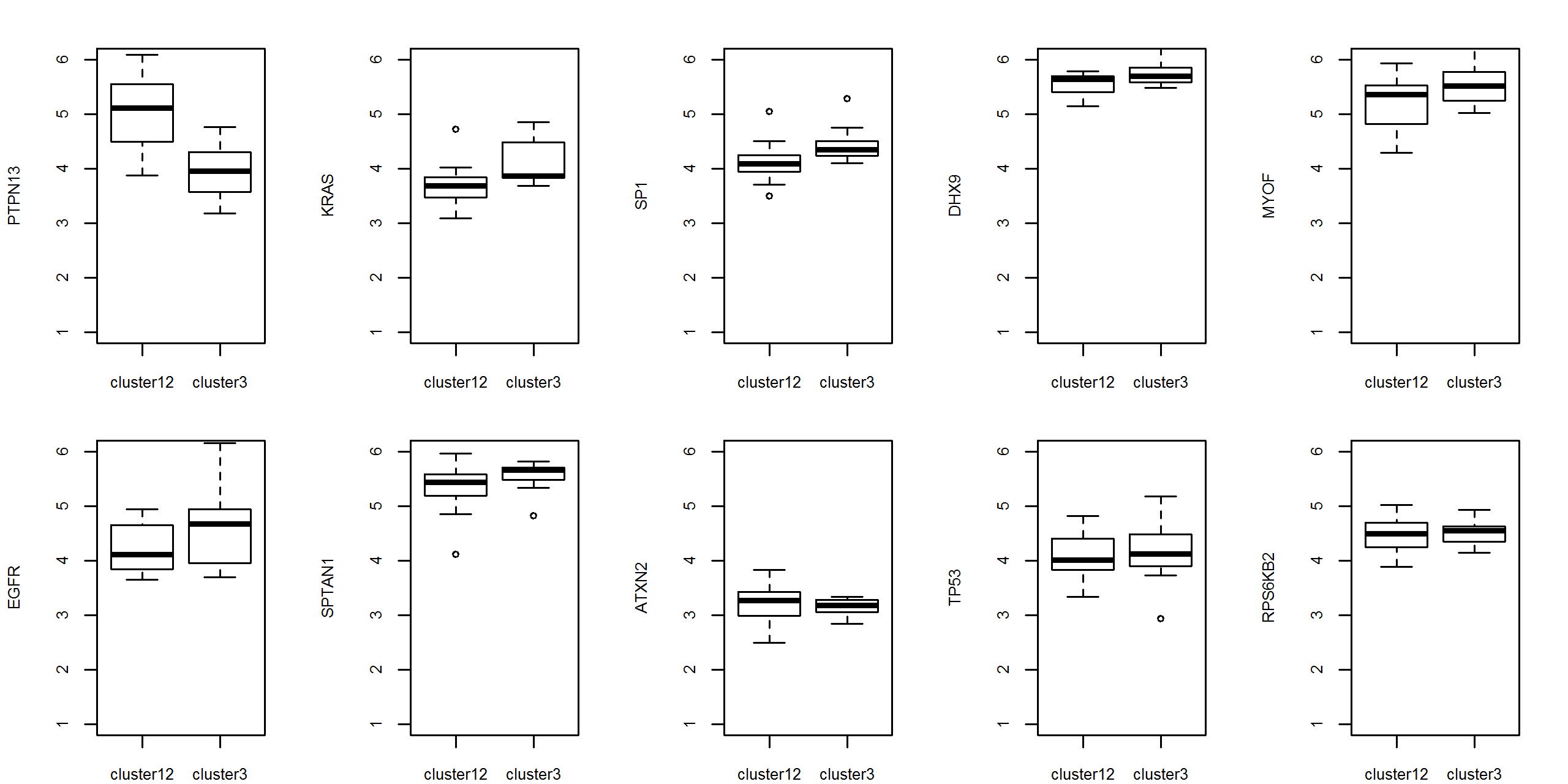

Supplement: Additional file 11 — 10 gene expression in tumors with good and bad outcome. The boxplot of expression of 10 genes with recurrent mutation in the tumors of clusters 1, 2 (good outcome) and cluster 3 (bad outcome), respectively. Y-axis is the log2 RPKM expression. [file 1755-8794-7-32-S11.tiff]

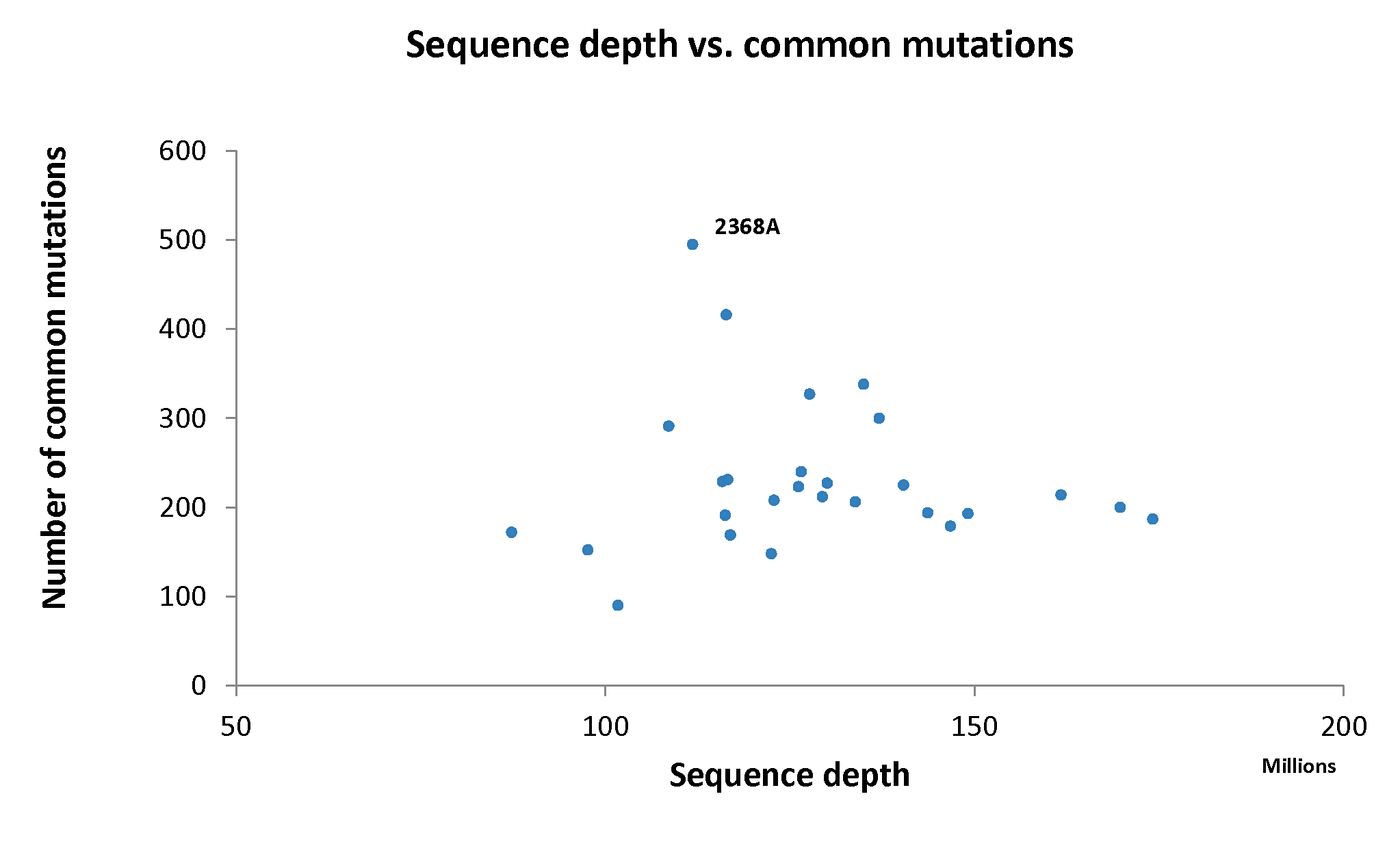

Supplement: Additional file 14 — Sequence depth vs. number of overlap mutations between DNA and mRNA. No correlation is observed and the tumor with the highest number of common mutations (Lu2368A) has much lower sequence depth than most of samples. X-axis is the minimum depth of tumor and normal from both exome-seq and mRNA-seq. Y-axis is the number of overlap mutations. [file 1755-8794-7-32-S14.png]

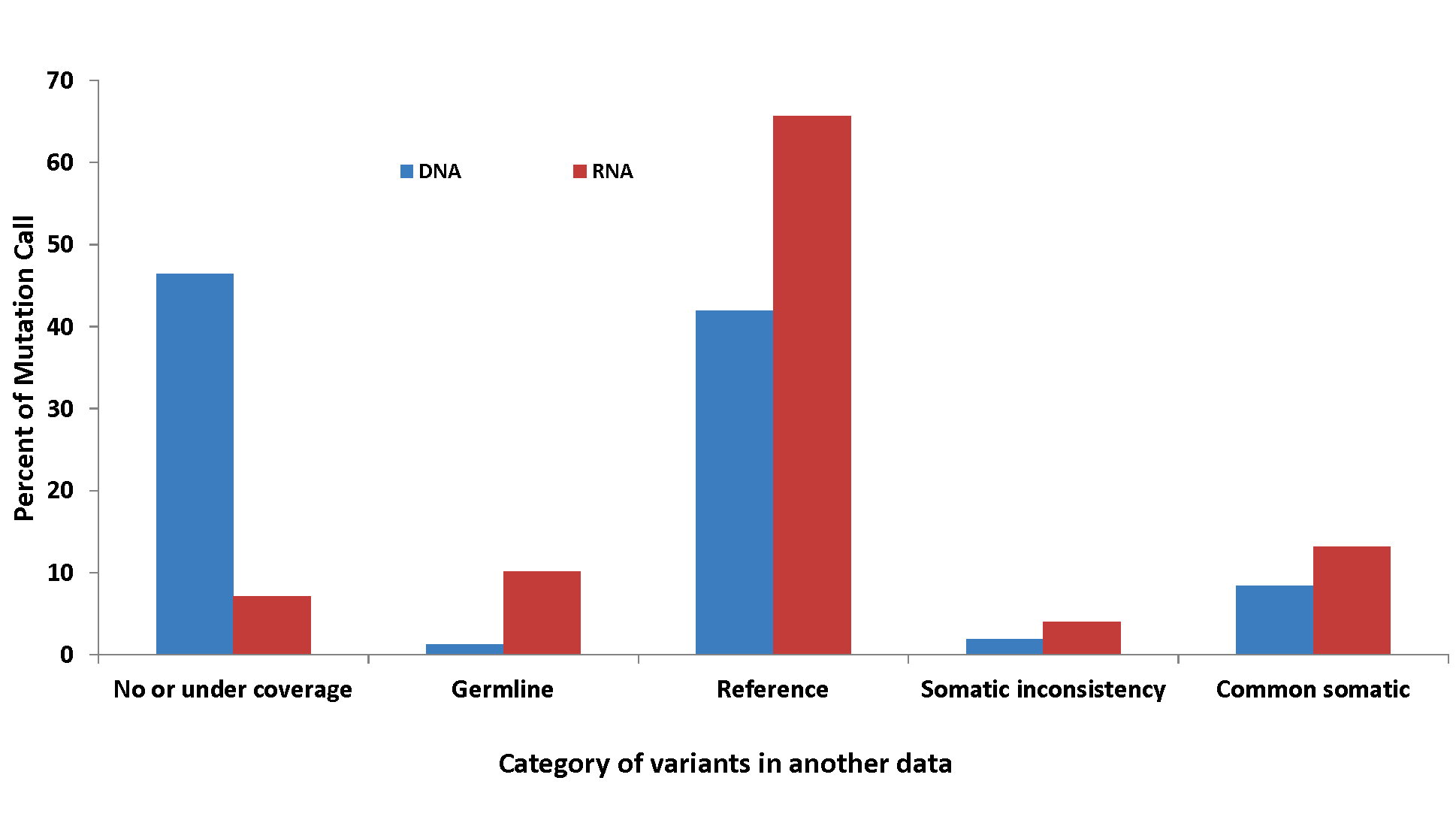

Supplement: Additional file 15 — Backfill information of mutation positions of one data in another. For the mutations called in DNA (or RNA) but not in RNA (or DNA), the coverage and allele information for the genomic positions in RNA (or DNA) are examined for potential genotype/mutation calls and tabulated by category. For DNA mutations not called in RNA, majority of them (46%) are lack of sufficient coverage (genes not expressed) while majority of mutations called in RNA but not in DNA (63%) do not have the mutation allele in the tumor DNA (or reference call). [file 1755-8794-7-32-S15.png]
