# Supplementary material for: Conserved recurrent gene mutations correlate with pathway deregulation and clinical outcomes of lung adenocarcinoma in never-smokers
Source: BMC Med Genomics. 2014 Jun 4;7:32. doi: 10.1186/1755-8794-7-32 (PMC4060138; doi:10.1186/1755-8794-7-32)
Supplement: Additional file 5 — Enriched pathways for the genes with overlap mutations. List of enriched pathways using the genes with mutations found in both DNA and RNA. [file 1755-8794-7-32-S5.doc]

**Additional file 5**: Significant pathways for the genes with recurrent mutations

|  | Ingenuity Canonical Pathways | -log(p-value) | Ratio (focus genes/total genes) | Molecules |
| --- | --- | --- | --- | --- |
| 1 | UVC-Induced MAPK Signaling | 4.43 | 1.43E-01 | BRAF,TP53,RRAS2,KRAS,PRKD3,EGFR |
| 2 | Thyroid Cancer Signaling | 3.49 | 1.19E-01 | BRAF,TP53,RRAS2,KRAS,CTNNB1 |
| 3 | UVA-Induced MAPK Signaling | 3.46 | 7.69E-02 | TP53,RRAS2,TIPARP,RPS6KB2,TNKS2,KRAS,EGFR |
| 4 | Telomerase Signaling | 3.2 | 7.07E-02 | TP53,PPP2R1A,RRAS2,SP1,HDAC10,KRAS,EGFR |
| 5 | Acute Myeloid Leukemia Signaling | 2.97 | 7.32E-02 | BRAF,MAP2K7,RRAS2,RPS6KB2,KRAS,PML |
| 6 | Hereditary Breast Cancer Signaling | 2.81 | 5.69E-02 | TP53,RRAS2,HDAC10,SLC19A1,KRAS,RFC1,SMARCA4 |
| 7 | RAN Signaling | 2.78 | 1.3E-01 | KPNB1,CSE1L,RANBP2 |
| 8 | Gap Junction Signaling | 2.68 | 4.65E-02 | RRAS2,TUBA1A,SP1,KRAS,CTNNB1,PRKD3,ADCY7,EGFR |
| 9 | CMP-N-acetylneuraminate Biosynthesis I (Eukaryotes) | 2.66 | 1.11E-01 | GNE,CMAS |
| 10 | PI3K/AKT Signaling | 2.62 | 4.93E-02 | TP53,PPP2R1A,JAK1,RRAS2,RPS6KB2,KRAS,CTNNB1 |
| 11 | GDP-glucose Biosynthesis | 2.48 | 1.18E-01 | PGM5,PGM2 |
| 12 | Melanoma Signaling | 2.44 | 8.7E-02 | BRAF,TP53,RRAS2,KRAS |
| 13 | Ovarian Cancer Signaling | 2.42 | 4.96E-02 | BRAF,TP53,RRAS2,RPS6KB2,KRAS,CTNNB1,EGFR |
| 14 | Glucose and Glucose-1-phosphate Degradation | 2.34 | 9.09E-02 | PGM5,PGM2 |
| 15 | IL-4 Signaling | 2.31 | 6.41E-02 | NFAT5,JAK1,RRAS2,RPS6KB2,KRAS |
| 16 | Protein Ubiquitination Pathway | 2.27 | 3.75E-02 | USP43,PSMA5,CUL1,HSPA13,FBXW7,PSMD1,NEDD4L,UBE3A,DNAJB5,USP48 |
| 17 | HER-2 Signaling in Breast Cancer | 2.23 | 6.25E-02 | TP53,RRAS2,KRAS,PRKD3,EGFR |
| 18 | CNTF Signaling | 2.2 | 7.69E-02 | JAK1,RRAS2,RPS6KB2,KRAS |
| 29 | Superpathway of Cholesterol Biosynthesis | 2.12 | 3.57E-02 | FDPS,ACAT2,LSS |
| 20 | Glycogen Degradation II | 2.12 | 1.33E-01 | PGM5,PGM2 |
| 21 | Endometrial Cancer Signaling | 2.11 | 7.02E-02 | TP53,RRAS2,KRAS,CTNNB1 |
| 22 | Colorectal Cancer Metastasis Signaling | 2.05 | 3.57E-02 | BRAF,TP53,JAK1,RRAS2,KRAS,CTNNB1,ADCY7,LRP1,EGFR |
| 23 | p70S6K Signaling | 2.04 | 4.69E-02 | PPP2R1A,JAK1,RRAS2,KRAS,PRKD3,EGFR |
| 24 | Role of CHK Proteins in Cell Cycle Checkpoint Control | 2.02 | 7.02E-02 | TP53,PPP2R1A,SLC19A1,RFC1 |
| 25 | Neuregulin Signaling | 2.01 | 5E-02 | RRAS2,RPS6KB2,KRAS,PRKD3,EGFR |
