# Supplementary material for: Conserved recurrent gene mutations correlate with pathway deregulation and clinical outcomes of lung adenocarcinoma in never-smokers
Source: BMC Med Genomics. 2014 Jun 4;7:32. doi: 10.1186/1755-8794-7-32 (PMC4060138; doi:10.1186/1755-8794-7-32)
Supplement: Additional file 9 — Differential expression between tumor and normal for the genes with recurrent mutations from edgeR. logFC – log2 fold change; FC – fold change; logCPM – normalized average expression across tumor and normal sample in log2 scale; PValue – differential p value between tumors and normal samples; FDR – false discovery rate. [file 1755-8794-7-32-S9.doc]

**Additional file 9 – The differential expression of 10 genes with recurrent mutations between tumor and normal**

| Symbol | logFC | FC | logCPM | PValue | FDR |
| --- | --- | --- | --- | --- | --- |
| SPTAN1 | -0.93585 | -1.91302 | 8.936804 | 3.23E-23 | 7.63E-22 |
| RPS6KB2 | 0.489368 | 1.403829 | 5.034478 | 9.08E-09 | 4.6E-08 |
| TP53 | 0.453189 | 1.369063 | 5.572406 | 4.58E-05 | 0.000132 |
| EGFR | 0.467424 | 1.382638 | 6.929959 | 0.00257 | 0.005298 |
| ATXN2 | -0.25853 | -1.19626 | 5.378874 | 0.003985 | 0.007911 |
| DHX9 | 0.240823 | 1.181667 | 7.720704 | 0.006101 | 0.011708 |
| SP1 | -0.20255 | -1.15073 | 7.230157 | 0.0268 | 0.044671 |
| MYOF | 0.242372 | 1.182936 | 8.013522 | 0.034436 | 0.055946 |
| KRAS | 0.213074 | 1.159155 | 6.120272 | 0.052819 | 0.081838 |
| PTPN13 | 0.082871 | 1.059124 | 7.757509 | 0.627377 | 0.695117 |
